# Breathing on chip: Dynamic flow and stretch accelerate mucociliary maturation of airway epithelium *in vitro*

**DOI:** 10.1016/j.mtbio.2023.100713

**Published:** 2023-06-27

**Authors:** Janna C. Nawroth, Doris Roth, Annemarie van Schadewijk, Abilash Ravi, Tengku Ibrahim Maulana, Christiana N. Senger, Sander van Riet, Dennis K. Ninaber, Amy M. de Waal, Dorothea Kraft, Pieter S. Hiemstra, Amy L. Ryan, Anne M. van der Does

**Affiliations:** a Hastings Center for Pulmonary Research, Division of Pulmonary, Critical Care and Sleep Medicine, Department of Medicine, University of Southern California, Los Angeles, CA, USA; b Emulate Inc., Boston, MA, USA; c Helmholtz Pioneer Campus and Institute for Biological and Medical Imaging, Helmholtz Zentrum München (GmbH), Neuherberg, Germany; d Chair of Biological Imaging at the Central Institute for Translational Cancer Research (TranslaTUM), School of Medicine, Technical University of Munich, Munich, Germany; e PulmoScience Lab, Department of Pulmonology, Leiden University Medical Center, Leiden, the Netherlands; f Department of Infectious Diseases, Leiden University Medical Center, Leiden, the Netherlands; g Department of Stem Cells and Regenerative Medicine, University of Southern California, Los Angeles, CA, USA

**Keywords:** Organs-on-chips, Airway epithelium, Mucociliary clearance, Airflow, Mechanobiology

## Abstract

Human lung function is intricately linked to blood flow and breathing cycles, but it remains unknown how these dynamic cues shape human airway epithelial biology. Here we report a state-of-the-art protocol for studying the effects of dynamic medium and airflow as well as stretch on human primary airway epithelial cell differentiation and maturation, including mucociliary clearance, using an organ-on-chip device. Perfused epithelial cell cultures displayed accelerated maturation and polarization of mucociliary clearance, and changes in specific cell-types when compared to traditional (static) culture methods. Additional application of airflow and stretch to the airway chip resulted in an increase in polarization of mucociliary clearance towards the applied flow, reduced baseline secretion of interleukin-8 and other inflammatory proteins, and reduced gene expression of matrix metalloproteinase (MMP) 9, fibronectin, and other extracellular matrix factors. These results indicate that breathing-like mechanical stimuli are important modulators of airway epithelial cell differentiation and maturation and that their fine-tuned application could generate models of specific epithelial pathologies, including mucociliary (dys)function.

## Graphical abstract

**Figure:**
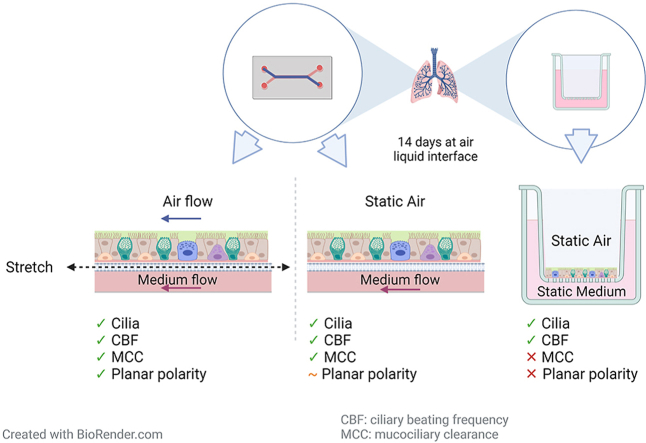

## 1 Introduction

The mucosal surface of the airways continuously removes contaminants from inhaled air by mucociliary clearance, which provides an essential defense mechanism against infection and/or damage of the lungs. This function relies on an ensemble of specialized epithelial cells that use motile cilia to transport secreted mucus, along with trapped airborne matter, out of the airways [1]. Although standard *in vitro* cultures of airway epithelial tissues feature all major cell types after three weeks of differentiation or earlier, a recent detailed examination of the mechanics of ciliary beat in such cultures showed that full maturity of ciliary beat and associated full maturity of mucociliary clearance may require more than 60 days of differentiation [2]. Moreover, these static culture conditions do not provide experimental control over epithelial differentiation towards the cellular composition characteristic of different airway branching generations. The cellular composition instead emerges based on the use of cell culture medium, donor, and culture protocol [3,4]. These limitations complicate translational value of (human) airway epithelial cell models, particularly when studying how infectious agents breach mature mucociliary clearance, or why some disease processes preferentially affect the large or the small airways [5]. It is currently unknown what mechanisms contribute to the tight regulation of mucociliary clearance during development and to homeostasis in the adult lungs.

In addition, breathing-related mechanical stresses, including stretch and shear stresses act on lung epithelial tissues throughout development and adult life *in vivo* [6], differ in magnitude in large versus small airways [7,8], and can improve airway epithelial barrier function *in vitro* [9]. Normal breathing creates a diverse mechanical landscape along the respiratory tree. Computational models suggest that breathing-associated cyclic tensile strain tends to increase as a function of branching generation, whereas the shear stress of breathing-associated airflow reduces [7,8]. *In vitro* alveolar epithelial tissue responds to these physiological cyclic strains by accelerated differentiation of specific cell types [10]. A pivotal study presenting the first alveolar Lung-on-Chip model in 2010 showed benefits for disease modeling in dynamically perfused and cyclically strained alveolar epithelial tissue cultures using cell lines [11]. However, due to a lack of human airway models in which such breathing forces can be mimicked, it remains unclear whether physiological breathing-related forces may also positively impact airway epithelial function. Traditionally used insert culture models are completely static, and the first generation of Airway-on-Chip models, despite other advancements such as perfusion, typically included rigid membranes [12,13], preventing the use of stretch, and lacked dynamic air flow. Here, we hypothesized that normal breathing-associated airflow shear and strain can modulate mucociliary function and development of human primary bronchial epithelial cells (hPBEC).

To test our hypothesis, we developed a robust protocol for differentiation of hPBEC at air-liquid interface (ALI) on a stretchable, porous membrane in an airway-on-chip device. Using this advanced cell culture system, we assessed mucociliary clearance function and differentiation of airway epithelium in response to a dynamic microfluidic environment with application of stretch and airflow shear stress levels that were set to mimic the small airway environment during normal tidal breathing.

## 2 Results

### 2.1 Optimization of airway epithelial cell culture on chip

**Fig. 1:**
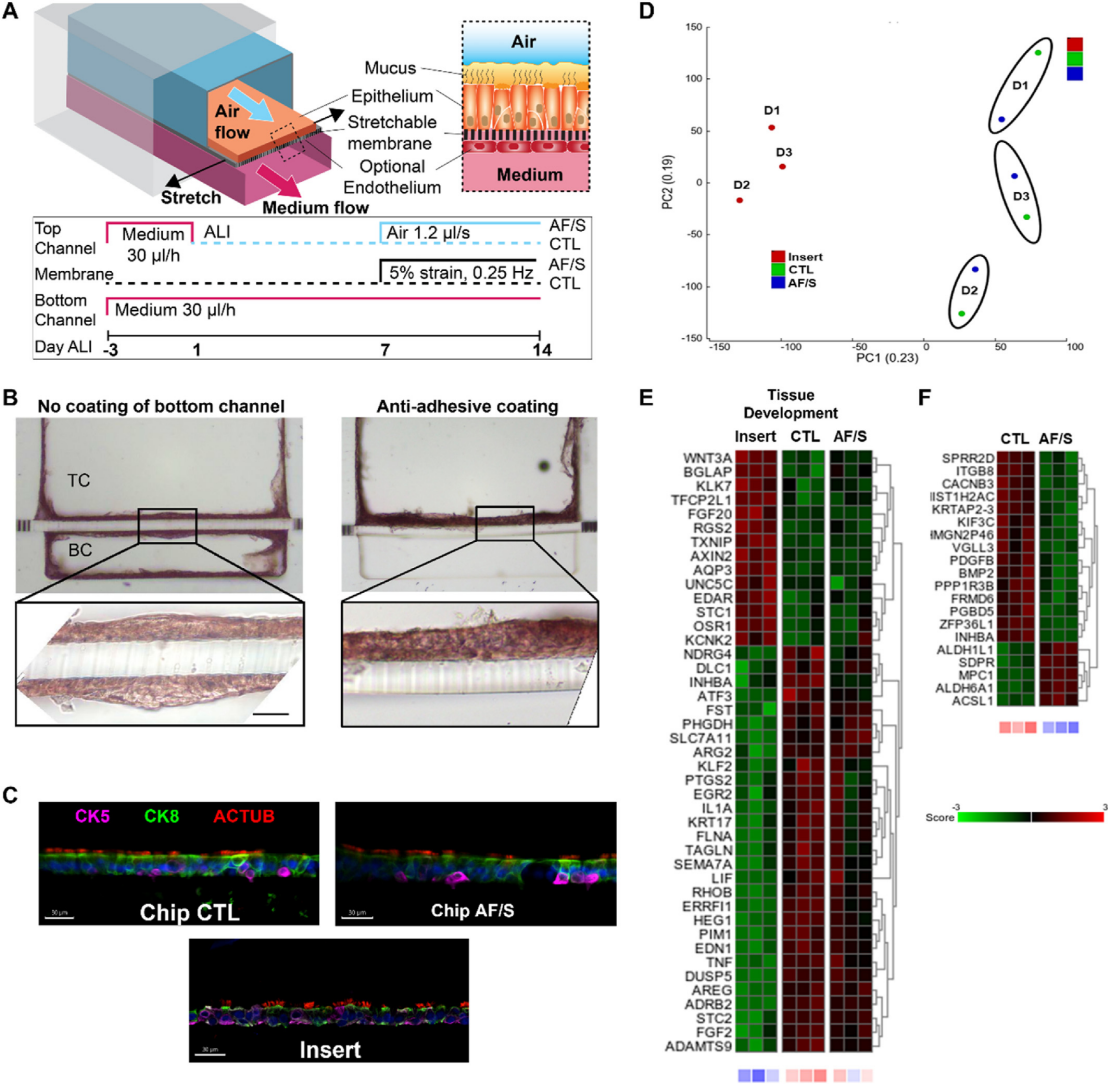
Dynamic chip culture skews cellular composition of the airway epithelium and accelerates maturation of mucociliary clearance in hPBECs. **A.** Airway basal epithelial cells are cultured in the top channel on a porous stretchable membrane. Endothelial cells can optionally be grown in the bottom vascular channel. Medium flow, air flow and membrane stretch are applied as indicated in the timeline. **B.** Histology cross-sections (H&E stain) of a fully invaded chip with hPBECs growing in both channels on the left, and a Pluronic-treated chip with no invasion of the bottom channel on the right. TC, top channel, BC, bottom channel. Zoom: cells line both sides of the membrane. Scalebar: 50 ​μm. **C.** Representative image of chip cross-section of a control (CTL) chip culture, an airflow and stretch-exposed (AF/S) chip culture and an insert culture with stainings for cytokeratin 5 (CK5, basal cells), −8 (CK8), and acetylated alpha-tubulin (ACTUB, cilia). Scalebar: 30 ​μm. **D.** PCA on all transcriptomes showing distinct clusters of airway epithelial cells from donors (N ​= ​3) in CTL chips, AF/S chips, or cell culture inserts coated with collagen IV. **E.** Heat map displaying the Z score of DEGs related to the tissue development pathway from Table 2, expression was compared between insert, CTL chip and AF/S chip cultures with N ​= ​3 donors that are paired (one donor per column). The Z scores for individual genes are represented in green and red, while the average Z scores (underneath the heat maps) of all genes in the gene set are represented in blue and red. **F.** Heat map displaying the Z score of all 21 DEGs between AF/S and CTL chip cultures. N ​= ​3 donors that are paired between AF/S and CTL (one donor per column). (For interpretation of the references to colour in this figure legend, the reader is referred to the Web version of this article.)

To test our hypothesis, we used the microfluidic Chip-S1® (Emulate Inc.), which features two adjacent, perfusable microchannels separated by a flexible and highly porous poly (dimethylsiloxane) (PDMS) membrane. hPBEC cultured on the apical side of the membrane can be exposed to air while interfacing with the (vascular) bottom compartment that is perfused with medium (Fig. 1A top and Supplementary Fig. 1A). The PDMS membrane is suited for dynamically stretching the epithelial tissue but was found to require extensive optimization of surface functionalization and cell culture protocol for successful hPBEC differentiation. Previously developed protocols for airway chips using cell-culture optimized, but rigid, membranes [12,13], failed to reliably support cellular adhesion on the PDMS substrate in this chip (data not shown). After testing a variety of options, which consisted mainly of various mixtures of collagen I, collagen IV, fibronectin and/or BSA or these compounds alone, we found that coating the activated membrane with high concentrations of collagen IV (300 ​μg/mL), followed by seeding hPBEC at high density (∼300,000 ​cells/cm^2^), led to robust cell adhesion and confluent monolayer formation in the top channel. We subjected the hPBEC to dynamic medium perfusion in both top and bottom channel during the submerged culture phase and next switched to air-liquid interface (ALI) in the top channel to initiate differentiation, while medium perfusion continued in the bottom channel (Fig. 1A bottom). The relatively large pore size of the membrane (7 ​μm) (Supplementary Fig. 1B) stimulated spontaneous and progressive transmembrane migration of hPBEC in the majority of donors, as verified with our newly developed method of chip sectioning (Fig. 1B left and Supplementary Fig. 1C and D). Since bottom channel invasion limited visibility and frequently led to degeneration and delamination of tissue in the top channel, we evaluated a variety of strategies to prevent epithelial cell migration. Chemical cell migration inhibitors and changes to the cell culture medium (Supplementary Table 1) proved to be ineffective or detrimental to differentiation (data not shown), gel barriers were effective but unreliable (Supplementary Fig. 1E and 7), and endothelial cells used as a barrier [14] would require co-culture with endothelial cells which was undesired for this study as it confounds the comparison to insert culture. We eventually identified a robust solution by functionalizing the bottom channel with the anti-fouling agent Pluronic F-127 [15]. Using this biocompatible and removable anti-adhesion coating, bottom channel invasion was significantly reduced for the cultures from all evaluated donors (Fig. 1B right and Supplementary Fig. 1F), enabling robust hPBEC differentiation as well as optional co-culture with primary endothelial cells at a time point of choice (Supplementary Fig. 2and Supplementary Videos 1 and 2) and as needed for applications involving vascular and immune cell responses [13].

Supplementary video related to this article can be found at https://doi.org/10.1016/j.mtbio.2023.100713

The following is/are the supplementary data related to this article:

Video 1

Video 2

### 2.2 Comparison between static insert and dynamic chip cultures

Next, to assess the effects of the microfluidic and flexible environment on airway epithelial development, we compared traditional, fully static cell culture inserts with the medium-perfused chip model (Table 1). We evaluated hallmarks of airway epithelial development: pseudostratification and presence of various epithelial cell types [16] at day 14 of ALI [17]. Both chip and insert cultures exhibited a classical pseudostratified layer with basal cells located close to the membrane and luminal cells with visible ciliation (Fig. 1C). We hypothesized that breathing-related mechanical cues may modulate airway epithelial development. To test this hypothesis, we added airflow and stretch to the chip culture and compared epithelial culture development between these airflow/stretch (AF/S) chips, control (CTL) chips with medium perfusion but static ALI, and fully static insert cultures (Table 1). The membrane of AF/S chips was actuated linearly and perpendicular to the channel at a rate of 0.25 ​Hz to achieve maximally 5% cyclic stretch in the center of the chip. This strain is the estimated physiological stretch in the small airways during tidal breathing at rest [7]. Airflow in AF/S chips was set to generate airflow shear stress of ca. 0.1 ​mPa on the cells, which is comparable to small airway conditions during tidal breathing [18].

**Table 1:** Overview of airway epithelial culture conditions.

Culture condition	Cell culture inserts	Medium-perfused chip (CTL-Chip)	Medium-perfused chip with airflow and stretch (AF/S-Chip)
**Properties**	• ALI • Fully static	• ALI • Static air • 30 ​μl/h medium flow	• ALI • 1.2 ​μl/s air flow • 30 ​μl/h medium flow • 5% membrane stretch at 0.25 ​Hz

**Table 2:** Top 10 Pathways associated with DEGs between CTL chip and insert cultures.

Pathway description b [# genes]	b DEGs	ID	p value	q value
Response to oxygen containing compound^a^ [1653]	43	GO:1901700	2.76 e^−22^	2.87 e^−18^
Signaling receptor regulator activity^a^ [543]	27	GO:0030545	4.15 e^−21^	2.16 e^−17^
Tissue development^a^ [1916]	44	GO:0009888	1.03 e^−20^	3.56 e^−17^
Regulation of cell population proliferation^a^ [1745]	41	GO:0042127	1.23 e^−19^	3.12 e^−16^
Molecular function regulator^b^ [1953]	43	GO:0098772	1.5 e^−19^	3.12 e^−16^
Locomotion^a^ [1921]	42	GO:0040011	5.59 e^−19^	9.7 e^−16^
Signaling receptor binding^b^ [1550]	38	GO:0005102	8.29 e^−19^	1.23 e^−15^
Regulation of multicellular organismal development^a^ [1394]	36	GO:2000026	1.53 e^−18^	1.99 e^−15^
Regulation of cell death^a^ [1643]	38	GO:0010941	5.73 e^−18^	6.62 e^−15^
Cell migration^a^ [1556]	37	GO:0016477	6.92 e^−18^	7.19 e^−15^

a Gene Ontology Biological Process.

b Gene Ontology Molecular Function.

We applied AF/S between day 7 and 14 of ALI (Fig. 1A bottom) to allow for barrier formation before the application of mechanical forces and to provide sufficient time for key cellular markers to change in expression during the course of the experiment [17]. We verified that lactate dehydrogenase (LDH) levels were similar between both chip groups, indicating comparable levels of cell viability (Supplementary Fig. 3A). To compare effects between the various culture conditions on tissue organization and MCC function in hPBECs, we performed an unbiased bulk RNA-sequencing (RNAseq) analysis on RNA isolated from hPBEC in static cultures (inserts) and dynamic cultures (CTL and AF/S chips) from three different donors collected at day 14 of ALI. Principal component analysis (PCA) revealed a pronounced difference between the transcriptional profiles of inserts and chip cultures, and a smaller difference between donors (Fig. 1D). We identified 182 differentially expressed genes (DEGs; q-value <0.05 and >1.5 fold change) between CTL chips and inserts (Supplementary Table 2) and performed pathway analyses (Table 2 with the Top 10 pathways depicted, heatmaps in Fig. 1E and Supplementary Fig. 4). Interestingly, one of the pathways with which the DEGs were associated was tissue development (Fig. 1E). Applying AF/S to the chip cultures shifted the transcriptional profile further. We identified 20 DEGs between AF/S chips and CTL chips (Fig. 1F, Supplementary Table 3). Further analysis did not reveal a specific pathway related to these genes; however, several genes related to TGF-β/BMP signaling (i.e., *BMP2*; *INHBA* and *PDGFB*) were reduced in expression in AF/S compared to CTL (Fig. 1F).

### 2.3 Perfused airway epithelial tissue cultures show accelerated maturation of mucociliary clearance

**Fig. 2:**
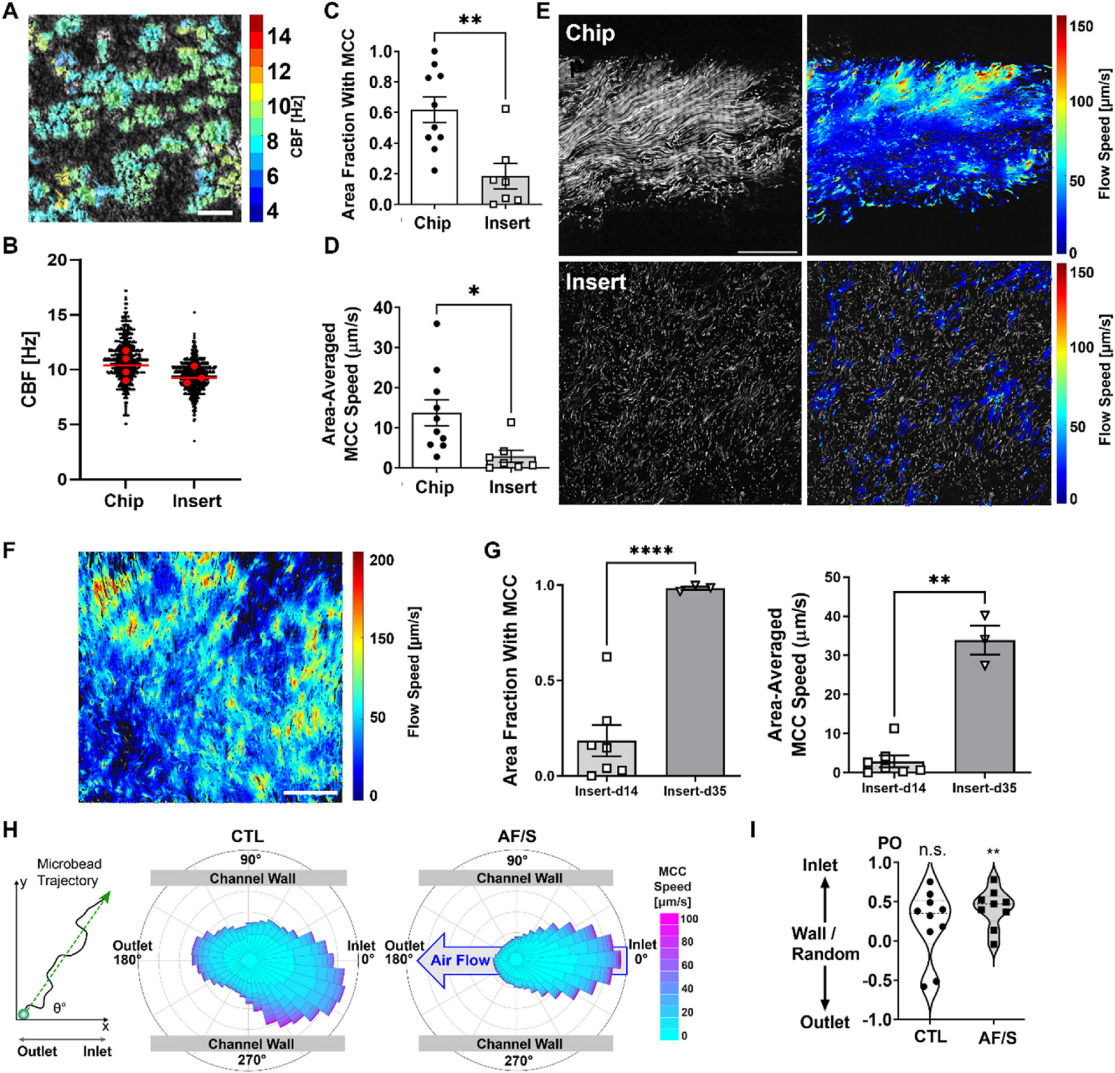
Exposure to airflow and stretch accelerates mucociliary clearance in hPBEC. **A.** Example of CBF heatmap (top; scalebar: 100 ​μm) and **B.** quantitative analysis comparing chip and insert (day 14 ALI) cultures. Data from N ​= ​2 donors, 2–4 chips or inserts per donor. Each black dot represents approximately 1 ciliated cell (chips: 2481 measurements; inserts: 2976 measurements). Red dots indicate means of individual inserts or chips and line indicates their median. **C.** Quantification of area fraction covered by MCC and **D.** area-averaged MCC speed (right). Depicted are mean ​± ​SEM of one chip or insert (day 14 ALI) per donor (chips N ​= ​9 donors; inserts: N ​= ​7 donors). ∗, p ​< ​0.05; ∗∗, p ​< ​0.01; two-tailed Welch's *t*-test. **E.** Example of fluorescent bead trajectories (left) and corresponding instantaneous flow speeds (right) in chip (top) and insert (bottom). Scale bar: 50 ​μm. **F.** Example of fluorescent bead flow speeds in insert at day 35 ALI. Scale bar: 200 ​μm. **G.** Quantification of area fraction covered by MCC (left) and area-averaged MCC speed (right) in inserts at day 14 ALI compared to day 35 ALI. Depicted are mean ​± ​SEM of 7 inserts at day 14 ALI from N ​= ​7 donors (one insert per donor) and 3 inserts from N ​= ​3 donors (one insert per donor)∗∗p ​< ​0.01, ∗∗∗∗p ​< ​0.0001; two-tailed Welch's *t*-test. **H.** From the overall transport direction of each fluorescent microbead (schematic on left), angle histograms of MCC direction for each condition are created, indicating the flow alignment relative to channel, relative to medium perfusion in bottom channel (from inlet to outlet), and relative to air flow in the AF/S top channel (large arrow, from inlet to outlet). CTL: 140,760 trajectories; AF/S: 119,696 trajectories. Data from N ​= ​9 (CTL) and N ​= ​8 (AF/S) donors, 1 chip per donor. **I.** Polar order (PO) parameter obtained from the bead trajectories in (e). The PO ranges from −1 to 1, with −1 indicating perfect polarization towards the outlet and 1 towards the inlet, and 0 is random flow or towards the wall. Each data point is PO from 1 chip per donor with N ​= ​9 (CTL) and N ​= ​8 (AF/S) donors. ∗∗, p ​< ​0.01 as assessed by 1-sample Wilcoxon signed rank test against null hypothesis (random or wall-bound flow with PO ​= ​0). (For interpretation of the references to colour in this figure legend, the reader is referred to the Web version of this article.)

Further exploring the potential changes in epithelial development, we turned to a central functional marker of mature airway epithelia: mucociliary clearance (MCC). High-speed videomicroscopy revealed a similar surface density and beat frequency of motile cilia in chip cultures compared to static inserts (Fig. 2A and B, and Supplementary Video 3). We next directly assessed MCC function by tracking the displacement of fluorescent microbeads by the ciliated epithelium. Despite the similar beating frequency, we observed markedly better MCC in chip cultures compared to insert cultures. We found that in chip cultures, a significantly higher proportion of the total surface area was cleared by MCC (MCC surface coverage), leading to twice the tissue-averaged MCC speed (Fig. 2C–E, Supplementary Video 4). It was previously shown that insert cultures lack efficient ciliary beat patterns and MCC at day 14 of ALI culture, and that this markedly improves at around day 30 of ALI [2]. We measured MCC in cell culture inserts at day 14 and day 35 and confirmed this improvement (Fig. 2F–G, Supplementary Video 4), ruling out any underlying issues with the insert cultures and instead indicating that MCC function was accelerated on chip. Together, these observations suggest that culturing hPBEC on a flexible membrane combined with dynamic flow provides critical environmental cues that promote functional maturation of multiciliated airway epithelium *in vitro*.

Supplementary video related to this article can be found at https://doi.org/10.1016/j.mtbio.2023.100713

The following is/are the supplementary data related to this article:

Video 3

Video 4

We next assessed ciliary beat activity and mucociliary clearance function after AF/S application. We found no significant difference in the density of motile cilia, CBF, average MCC speed, or MCC coverage (Supplementary Fig. 3C to E) between the two chip conditions. We noted that MCC in all chips tended to be aligned along the length of the channel and point towards either end of the chip. Interestingly, while MCC in CTL chips only showed a weak bias towards the inlet, MCC in AF/S chips was commonly directed towards the inlet, i.e., against the direction of the applied air and medium perfusion, as seen in the angle histograms of the fluorescent microbead trajectories for each condition (Fig. 2H). We quantified the polarization of MCC towards either chip inlet or outlet by using a normalized index of direction, the polar order parameter (PO). The PO analysis confirmed unidirectional MCC towards the inlet in AF/S chips, but not in CTL chips, when compared to the null hypothesis of random or perpendicular flow direction (Fig. 2I). Together, our observations suggest that physiological levels of airflow and stretch may have profound effects on *in vitro* airway epithelial ciliary maturation.

### 2.4 Dynamic airflow and cyclic strain changes VANGL1 expression related to multiciliated cell maturation

**Fig. 3:**
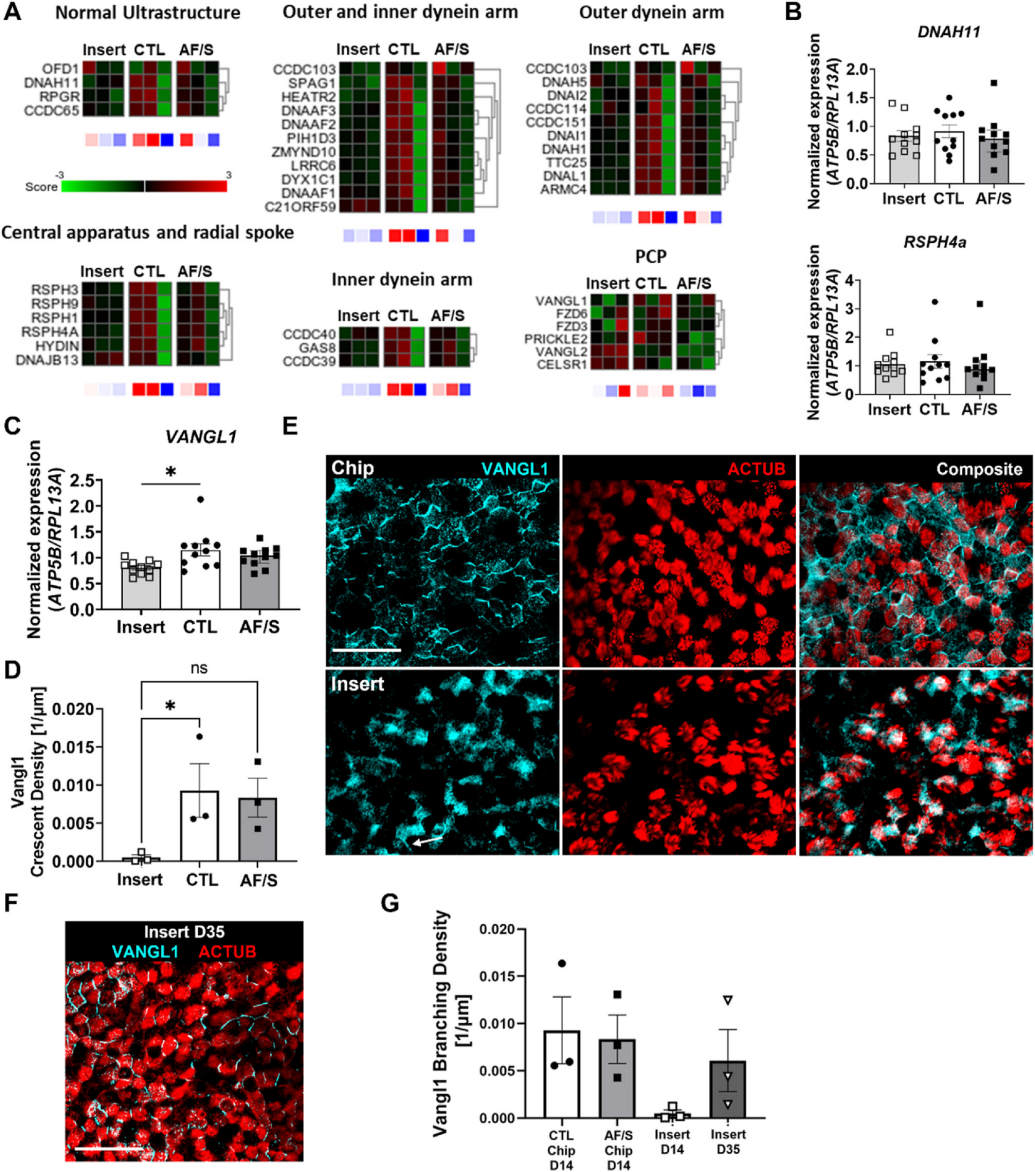
Culture on chip promotes VANGL1 expression **A.** Heat maps displaying the Z score of PCP genes between insert, CTL chip and AF/S chip cultures with N ​= ​3 donors that are paired between insert, CTL chip and AF/S chip cultures (one donor per column) **B.** Gene expression at day 14 ALI of *DNAH11* and *RSPH4a* for cultures on inserts (light gray bars; open squares), chips (open bars; black circles) or chips exposed to AF/S (dark gray; open circles); N ​= ​8–11 donors, one insert or chip per donor. Data are depicted as mean with SEM. **C.** gene expression at day 14 ALI of *VANGL1* for cultures on inserts (light gray bars; open squares), chips (open bars; black circles) or chips exposed to AF/S (dark gray; open circles); N ​= ​8–11 donors, one insert or chip per donor. Data are depicted as mean with SEM. **D.** Quantification of VANGL1 crescent density in CTL and AF/S chips and inserts at day 14 ALI. N ​= ​3 donors; each point is mean crescent density from multiple FOVs in 1 chip per donor. ∗, p ​< ​0.05 as assessed by one-way paired ANOVA with Dunnett's multiple comparison test. E. Representative IF staining of VANGL1 at day 14 ALI. Distinct VANGL1 crescents line most ciliated cells in chips of either condition (shown here: CTL). In contrast, static inserts of the same donor exhibit diffuse VANGL1 expression in most cells and very few such crescents (arrows). Scalebar 25 ​μm. **F.** Representative staining of VANGL1 at day 35 ALI in same donor as in Fig. 3 at day 14 ALI. Scale bar: 50 ​μm. **G.** Quantification of VANGL1 crescent density in chips at day 14 ALI compared to inserts at day 14 ALI and day 35 ALI. N ​= ​3 donors, one chip or insert each; each point is mean value from 1 insert or chip; columns and error bars represent mean ​± ​SEM.

To probe the mechanisms behind the accelerated MCC maturation and polarization in dynamic chip culture conditions (Fig. 1, Fig. 2), we analyzed the expression of genes related to cilia function, e.g. dyneins, in the RNAseq data. Compared to static inserts, chip cultures from two out of three donors showed increased expression levels in this gene set (Genes in Fig. 3A are grouped based on literature [19]). Follow-up qPCR analysis of a selection of these genes using a larger number of donors revealed however no significant changes in the dynein-related genes *DNAH11* and *RSPH4a* (Fig. 3B). We next assessed expression of planar cell polarity (PCP) genes and found that expression of the PCP gene *VANGL1*, which plays an important role in multiciliated cell maturation [2,20], was significantly increased in CTL chips compared to inserts (Fig. 3C). We further investigated VANGL1 on protein level. IF staining at day 14 ALI showed that localization of VANGL1 in both chip conditions was asymmetric and appeared in a crescent shape along boundaries of ciliated cells, whereas expression was diffuse with few crescent shapes in insert cultures of same age (Fig. 3D and E) but became comparable to chips at day 35 ALI (Fig. 3F–G). Asymmetric, crescent-like PCP localization to cell boundaries is a key indicator of functional maturation of ciliary beat in airway epithelia [2,20]. Together, these results support that the enhanced MCC functionality on chip might be promoted by accelerated maturation of multiciliated cells.

### 2.5 Airway epithelial cell composition in static and dynamic conditions

**Fig. 4:**
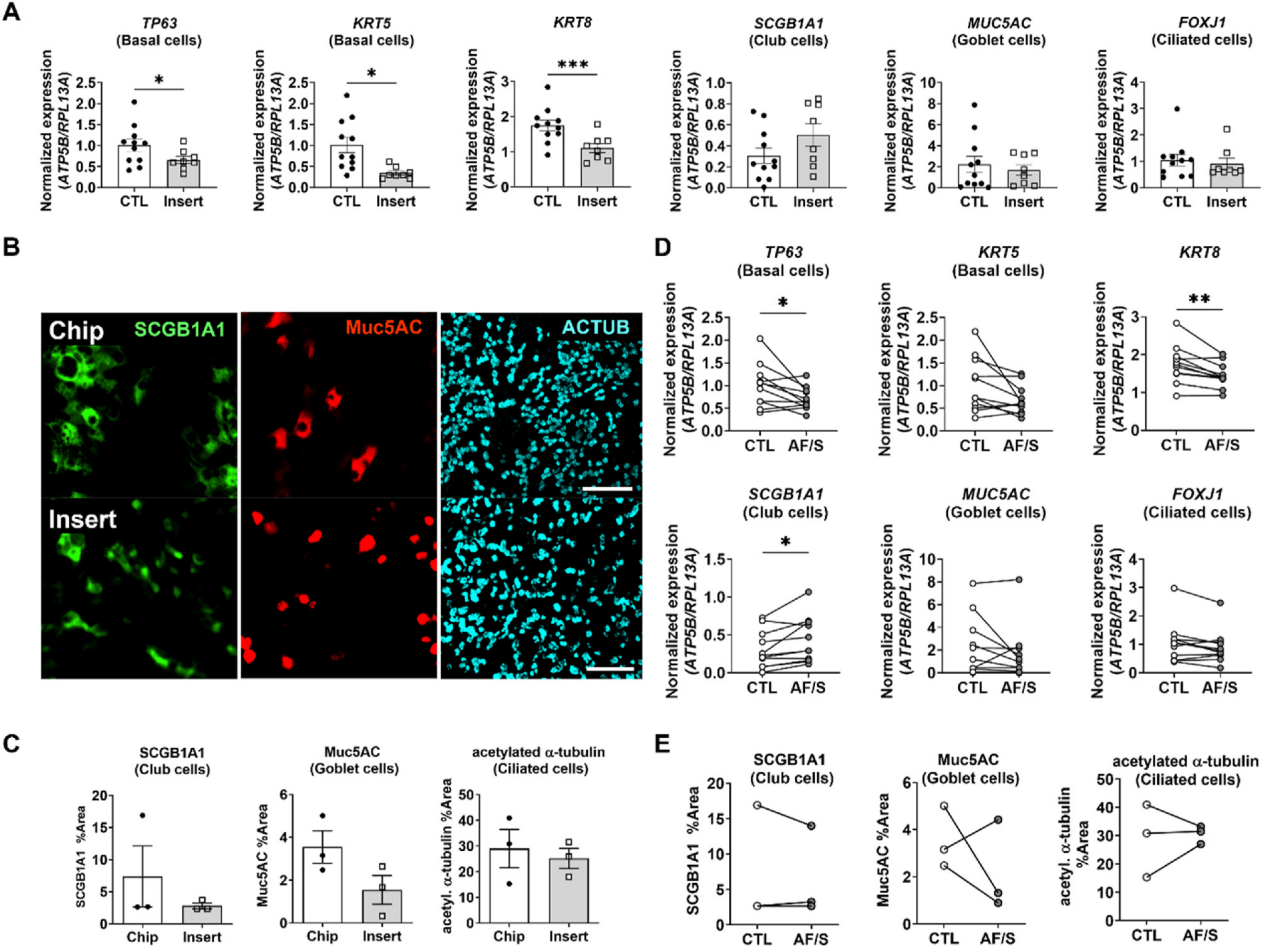
Effects of dynamic chip culture on epithelial cell differentiation **A.** Gene expression at day 14 ALI of *TP63* (basal cells), *KRT5* (basal cells)*, KRT8* (differentiated non-basal cells), *SCGB1A1* (club cells), *MUC5AC* (goblet cells) and *FOXJ1* (ciliated cells). Open circles: CTL chips; gray circles: AF/S-exposed chips; N ​= ​8 donors, one chip per donor. Data are depicted as mean with SEM. ∗, p ​< ​0.05; ∗∗p ​< ​0.01 as assessed by a two-tailed paired *t*-test. **B.** Representative IF staining of club (SCGB1A1), goblet (Muc5AC), and ciliated cells (acetylated alpha-tubulin, ACTUB) in chip and insert. Scalebar: 100 ​μm. **C.** Quantification of the relative surface area labeled with IF markers for each cell type. N ​= ​3 donors; each point is mean relative surface area labeled in multiple FOVs in 1 chip per donor. N.S. as assessed by Wilcoxon matched-pairs signed rank test. **D.** Gene expression of *TP63* (basal cells), *KRT5* (basal cells), *KRT8*, *SCGB1A1* (club cells), *MUC5AC* (goblet cells) and *FOXJ1* (ciliated cells) in collagen IV (COLIV)-coated chips and inserts at day 14 ALI. Data are shown as target gene expression normalized for the geometric mean expression of the reference genes *ATP5B* and *RPL13A*; N ​= ​8–11 donors, one chip or insert per donor. Data are depicted as mean ​± ​SEM. ∗, p ​< ​0.05; ∗∗∗, p ​< ​0.001, a paired two-tailed paired *t*-test. **E.** Quantification of the cell culture surface area fraction positive for IF markers of each cell type. Depicted are mean ​± ​SEM from N ​= ​3 donors with 3–6 chips or inserts each. No statistical difference was detected using one-tailed *t*-test.

To investigate if the increased mucociliary clearance was accompanied by changes in epithelial cell-type numbers, we assessed cellular composition via gene expression of cell-type marker genes and by immunohistochemistry. Compared to insert cultures, cell cultures in the chip exhibited significantly higher gene expression levels of basal cell markers (*TP63* and *KRT5*) and of keratin 8 (*KRT8*) (Fig. 4A). *KRT8* is a marker gene that is used for describing a population of cells that is variously listed as non-basal [21], luminal [21,22], (suprabasal) early progenitor [23] or basal luminal precursor [24] cells. Due to this lack of consensus in literature, we will address them as KRT8^+^ cells. Expression of differentiated luminal cell markers for secretory club cell (*SCGB1A1*), goblet cell (*MUC5AC*) and ciliated cells (*FOXJ1*) was not different from insert cultures (Fig. 4A). IF-staining of cell type-specific proteins confirmed similar levels of differentiated luminal cell in chip compared to insert cultures (Fig. 4B and C).

Compared to CTL chips, AF/S chips expressed significantly lower levels of genetic markers for basal cells (*TP63* and trend for *KRT5* (p ​= ​0.055)) and for KRT8^+^ cells. Expression of *SCGB1A1,* a marker of club cells, was significantly increased (Fig. 4D). The difference in gene expression may capture an early stage in club cell development as the tissue-level density of immunofluorescent (IF) labeled club cell protein was similar between groups (Fig. 4E and Supplementary Fig. 3B).

Together, these results indicate that the luminal cellular composition of the epithelium was not dramatically changed in chip cultures compared to inserts, despite a significant improvement in mucociliary functioning.

### 2.6 Dynamic airflow and cyclic strain reduce expression of inflammation and extracellular matrix-related genes and protein levels in airway epithelial cultures

To assess if other processes were affected by dynamic chip culture compared to static insert culture, we assessed markers of inflammation and related to extracellular matrix (ECM) production.

**Fig. 5:**
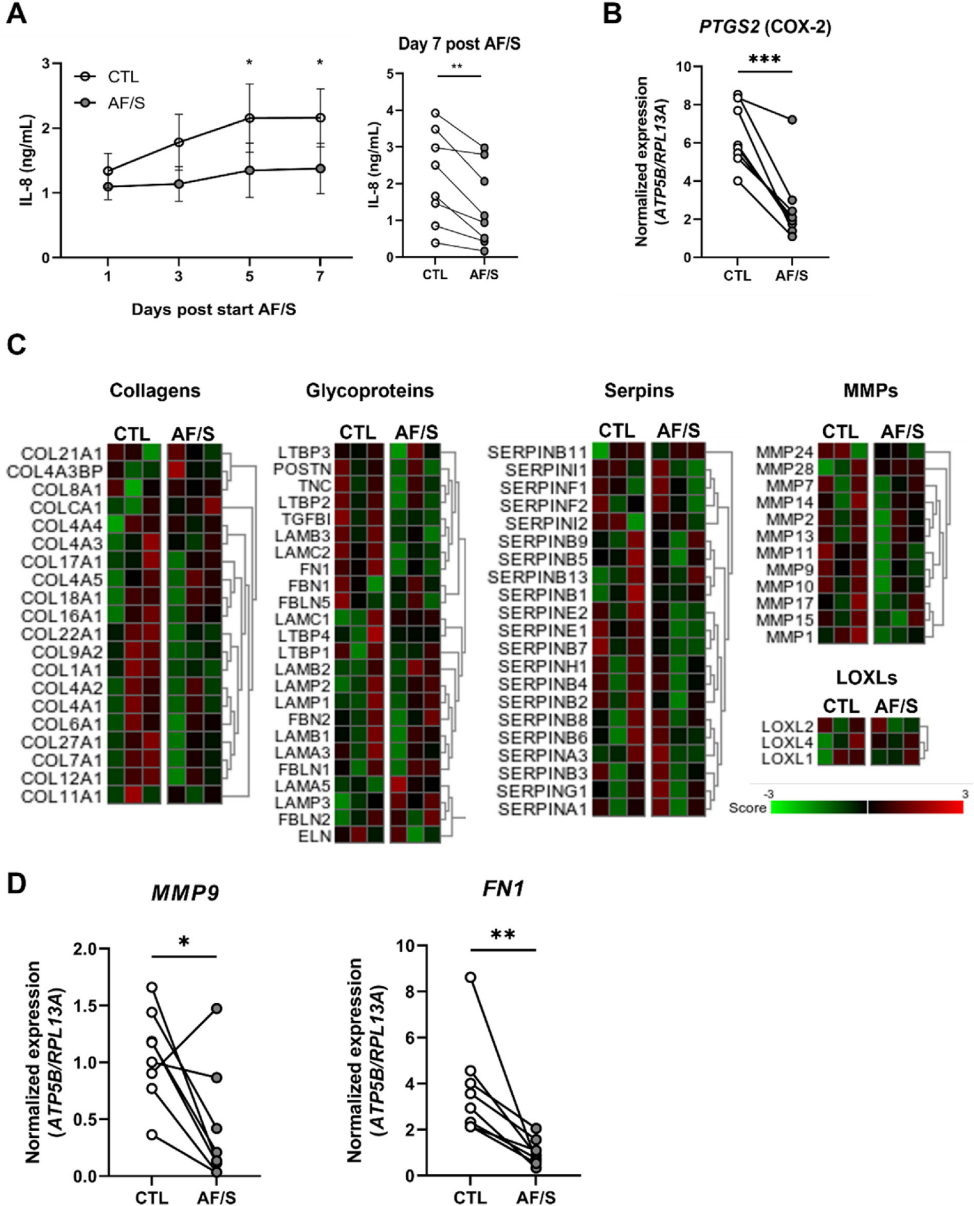
Exposure to airflow and stretch reduces baseline pro-inflammatory and ECM-related gene expression. **A.** Left: IL-8 protein levels (ELISA) in the basal channel medium collected 24 ​h after each medium change at the indicated times. Filled circles are CTL chips, open circles are AF/S chips; N ​= ​8 donors with 2 chips per donor, except for 2 donors with 1 AF/S chip each. Right: paired IL-8 levels at day 14 post ALI and 7 days of application of AF/S. ∗, p ​< ​0.05 as assessed by Mixed-effects analysis combined with a Sidak's multiple comparisons test. ∗∗, p ​< ​0.01 as assessed by paired *t*-test. **B.** Gene expression of *PTGS2* at day 14 ALI. Open circles: CTL chips, gray circles: AF/S-exposed chips; N ​= ​8 different donors, one chip/donor. Data are depicted as mean ​± ​SEM. ∗∗, p ​< ​0.01 as assessed by paired *t*-test. **C.** Heat map of Z-scores for ECM-related gene expression (RNAseq), i.e., collagens, glycoproteins, matrix metalloproteinases (MMPs), SERPINs and LOXLs, in AF/S and CTL chips at day 14 of ALI. RNA was derived from N ​= ​3 donors that are paired between AF/S and CTL (one column per donor)), 1 chip/donor. The Z scores for individual genes are represented in green and red. **D.** Gene expression assessed by qPCR analysis of *MMP9* and *FN1* at day 14 of ALI, Open circles: CTL chips, gray circles: AF/S chips.; ∗p ​< ​0.05; ∗∗, p ​< ​0.01 as assessed by a paired *t*-test, N ​= ​8 donors, one chip per donor. (For interpretation of the references to colour in this figure legend, the reader is referred to the Web version of this article.)

Baseline levels of IL-8, a typical inflammatory chemokine, were significantly lower in chip cultures at five and seven days after exposure to airflow and stretch (Fig. 5A). Expression of another pro-inflammatory marker, *PTGS2,* was also significantly lower in these cultures (Fig. 5B), as were protein levels of several cytokines (G-CSF, IL-9 and MIP-1β) as assessed by a multiplex assay, confirming an overall lower baseline level of inflammatory mediator production by the epithelial cultures exposed to airflow and stretch (Supplementary Fig. 5A).

To explore effects on ECM production, we assessed expression of collagens, glycoproteins (selection), serpins, MMPs, and Lysyl oxidase homologs (LOXLs)-related genes in our RNAseq data set (Fig. 5C). No overall change in expression in any of these groups was observed; however, expression of specific components, including *MMP1*, *MMP9*, *COL1A1* (collagen type-1) and *FN1* (Fibronectin), was downregulated in AF/S compared to CTL (supplementary Fig. 5B). qPCR analysis of *MMP9* and *FN1* for N ​= ​8 donors supported a significant downregulation of both these genes in AF/S chips compared to CTL chips (Fig. 5D). Together, these results suggest that physiologically relevant mechanical cues may influence inflammatory signaling and ECM-remodeling in airway epithelial tissues.

## 3 Discussion

Primary human airway epithelial cells differentiated in organ chips exhibited a notably stronger mucociliary clearance compared to static insert cultures of same age. MCC function depends on several factors, including mucus rheology, ciliated cell density, ciliary beat frequency, and ciliary beat waveform, such as cilia alignment, stroke trajectories, and temporal coordination [2,[25], [26], [27]]. Our data demonstrate that the density of ciliated cells and their beat frequencies were similar in all conditions, suggesting that these factors were not majorly contributing to the pronounced differences in MCC.

Instead, we posit that the chip tissues experienced accelerated maturation of ciliary beat properties, such as force generation and stroke pattern, as supported by the increased expression of PCP genes and localization of PCP proteins in chips compared to inserts. PCP protein localization is one of the hallmarks of multiciliated cell maturation and determines beat polarity [20]. The comparatively slower maturation of MCC in static inserts matches the timelines previously reported for such cultures, and these studies also showed that, while ciliary beat was detectable after 1 or 2 weeks at ALI, ciliary beat properties took many more weeks to mature until efficient mucus propulsion was observed [2]. Another study showed that beat frequency was regulated by an internal timing mechanism and not a geometric switch point (i.e. a waveform-dependent mechanism), and force was independent of frequency [28], confirming that beat frequency is neither an indicator of applied force nor the presence of a mature waveform.

Another effect we observed on chip was that MCC tended to oppose the direction of the applied perfusion in the bottom channel, an effect that was amplified when we applied additional airflow in the same direction in the top channel, indicating an impact on multiciliated cell polarity and organization in the total culture [29]. By contrast, a recent study showed that human induced pluripotent stem cell (hiPSC) derived airway epithelium generated MCC in the same direction as imposed flow [30]. Hence, different mechanisms may be implicated in determining ciliary beat polarity of primary epithelial cultures versus hiPSC-derived airway epithelial cultures, which may be related to the notion that hiPSC-derived cultures are sometimes considered to be relatively immature. It is tempting to speculate that this effect of airflow on directionality of MCC helps in directed movement of mucus towards the oral cavity, where it can be removed by swallowing or expectoration. This may occur despite the fact that airflow is bidirectional, since flowrates are higher during inspiration than during expiration. It may furthermore help to explain impaired MCC in e.g. patients with bronchiectasis, a condition with abnormal widening of the airways resulting in reduced airflow.

Importantly, our new chip culture protocol requires less time to achieve physiologically relevant MCC function compared to insert cultures. A variety of factors differ between chip and insert cultures that may have contributed to the accelerated maturation of PCP expression and mucociliary clearance on chip. The chips include a stretchable PDMS membrane with lower stiffness compared to the rigid PET membrane of the inserts. Substrate stiffness influences many cellular responses [31] but the airway epithelium remains understudied in this context and effects on mucociliary clearance have so far not been reported to our knowledge. In addition, a higher turnover of the medium could have provided the cell cultures with exposure to more nutrients (and less waste products) compared to insert models. Finally, increased evaporation under airflow may have increased mucus viscosity which could influence ciliary alignment [25].

Besides effects on ciliary maturation, these factors may also influence epithelial differentiation.

Rat tracheal epithelial cells cultured on four different platforms exhibited significant differences in cellular composition that mimicked either the proximal or distal airways, probably promoted by many factors that varied between platforms, including substrate stiffness and chemical composition, 2D and 3D substrate geometries, and differentiation duration [32]. In our chip cultures, we observed significant changes in gene expression related to the basal cell compartment compared to insert cultures but no major impact on gene expression related to the luminal cell population was observed. Perhaps the time frame of 7 days was too short to measure effects on changes in cellular composition. In future studies of such kind, it will also be important to investigate the role of individual factors in shaping differentiation, maturation, and epithelial functioning, such as mucociliary clearance, since we show significant functional differences despite finding only moderate changes in (basal) cell composition. The methods we developed will facilitate the modeling of different lung tissue compartments *in vitro* and enable future studies into the mechanisms by which mucociliary clearance is impaired in disease or upon damage, and whether this can be reversed by application of targeted mechanical cues or related pathways.

In this study we used human primary cells from multiple human donors (Supplementary Table 4) and studied differentiation in the presence of mechanical stimulation in order to capture robust, longer-term responses rather than acute insults or cell-line-specific behavior. We did not co-culture endothelial cells in the vascular compartment of the chip in this study as this would confound the comparison with inserts without endothelial co-culture and potentially mask the effects of biomechanical cues on airway epithelial biology. It would be interesting to study in the future how the changes in epithelial cellular composition and/or function affect the mesenchymal and the vascular compartment. Although we did not explore the isolated effects of dynamic flow and stretch on airway epithelial biology, the ability to deliver these cues and achieve measurable effects on mucociliary functioning using a commercially available chip model combined with a robust protocol for primary airway culture opens new avenues for investigating ciliogenesis, airway disease and epithelial regeneration in the presence of mechanical stresses and establish beneficial versus adverse effects. Our method could also be applicable to the differentiation of alveolar or airway cells derived from induced pluripotent stems cells [30,33] as well as multi-tissue airway models and enhance their structural and functional maturation *in vitro*.

## 4 Conflict of interest disclosure

J.N. is a former employee of Emulate, Inc. A.D. was supported by a Global 10.13039/501100000654Marie Curie fellowship (No. 748569) that included a 1-yr visit (2018–2019) at Emulate Inc. to work on their Lung-Chips. D.R. and T.M. performed their internship at Emulate Inc in 2018 and 2019, respectively. Materials from Emulate Inc. related to this work were therefore provided by Emulate Inc. The remaining authors have no conflicts of interest relevant to this publication.

## 5 Methods

### 5.1 Primary human bronchial epithelial cell sourcing and expansion

Here and in following steps, we report the procedures optimized in parallel in laboratories at LUMC, USC, and Emulate Inc. And specify differences in material and protocol choices as applicable. For donor characteristics see Supplementary Table 4.

#### 5.1.1 Leiden

Primary human bronchial epithelial cells (PBEC) were isolated from tumor-free resected bronchus rings obtained from lung cancer patients undergoing a resection surgery at the Leiden University Medical Center (LUMC, Leiden, the Netherlands). Patients from which this lung tissue was derived were enrolled in the biobank via a no-objection system for coded anonymous further use of such tissue (www.coreon.org). Within this framework, individual written informed consent is not needed. Since 01-09-2022, patients are enrolled in the biobank using active informed consent in accordance with local regulations from the LUMC biobank with approval by the institutional medical ethical committee (B20.042/Ab/ab and B20.042/Kb/kb). hPBEC were thawed in a T75 flask coated with 30 ​μg/mL bovine type-1 collagen (PureCol®, Advanced BioMatrix), 10 ​μg/mL fibronectin (Promocell, PromoKine, Bio-connect) and 10 ​μg/mL BSA (ThermoFisher Scientific) in BEpiCM-b basal medium (ScienCell Research Laboratories, Sanbio), supplemented with 100 U/mL penicillin (Lonza), 100 ​μg/mL streptomycin (Lonza) and bronchial epithelial cell growth supplement (ScienCell). When reaching ∼90% confluency, cells were trypsinized in 0.03% (w/v) trypsin (ThermoFisher Scientific), 0.01% (w/v) EDTA (BDH, Poole, UK), 0.1% glucose (BDH) in phosphate buffered saline (PBS) and prepared for seeding.

#### 5.1.2 USC

Fresh hPBEC were isolated from lung explant tissue from rejected donor transplant from subjects with no prior evidence of chronic lung disease with IRB approval from the University of Southern California (IRB# USC HS-18-00162) using established protocols [34]. Next, hPBEC were thawed in T75 flasks or 100 ​mm cell culture dishes coated with PureCol (Advanced Biomatrix, Carlsbad, CA, USA) using complete BEpiCM-b basal medium as described above. When reaching ∼90% confluency, cells were dissociated with Accutase (Sigma Aldrich, St. Louis, MO, USA) and prepared for seeding.

#### 5.1.3 Emulate Inc

Hydrogel experiments were performed with hPBECs from a commercial supplier (Lifeline Cell Technologies, Frederick, MD, USA) that were cultured similar to the methods stated for LUMC/USC.

### 5.2 Chip functionalization and cell culture

#### 5.2.1 Chip membrane activation

To hydrophilize the PDMS membrane, top and bottom channels of the Chip-S1® (Emulate, Inc., Boston, MA, USA) were filled with ER-1 solution (1 ​mg/mL in ER-2; Emulate Inc) followed by 10 ​min of UV-light exposure using a 36-Watt UV-chamber (NailStar Professional, Model NS-01-US). Next, channels were washed twice with ER-2 (Emulate Inc.). This ER-1/ER-2 procedure was repeated once more. After the second ER-2 wash, channels were washed and filled with cold PBS.

#### 5.2.2 Chip membrane coating

The chip top channel was emptied and subsequently completely filled with 300 ​μg/mL human collagen IV (Sigma) solution in PBS (approximately 35 ​μl). Chips were placed in petri dishes containing a small, open container of PBS to promote humidity and kept overnight in the incubator at 37 ​°C/5%CO_2_. For optimization experiments, also other coating components and their mixtures were used: 0.1% BSA (Gibco), 30 ​μg/mL bovine type-1 collagen (PureCol®, Advanced BioMatrix), or human fibronectin (Sigma). We found that BSA and collagen IV coating both resulted in reliable cell adhesion; however, collagen IV coated chips exhibited significantly better viability post seeding and significantly higher ciliation compared to BSA coated chips at day 14 ALI (Supplementary Fig. 6).

#### 5.2.3 Epithelial cell seeding and submerged culture on chip

The coating solution in the top channel was removed and replaced with so-called B/D complete medium: a 1:1 mixture of BEpiCM-b and DMEM medium (STEMCELL Technologies), supplemented with Bronchial Epithelial Cell Growth Supplement (ScienCell), and additional 50 ​nM EC-23 (Tocris); 25 ​mM HEPES (Cayman Chemical), 100 U/mL penicillin and 100 ​μg/mL streptomycin (ScienCell). B/D complete was pre-filtered using a 70 ​μm cell strainer (BD Biosciences) or a 0.22 ​μm vacuum filter unit (Steriflip™; Millipore, Sigma) to remove any suspended particles that could block flow in the Chip-S1®. Epithelial cells were seeded in B/D complete in the top channel at 3 ​× ​10^6^ ​cells/mL (ca. 90 ​K cells per chip, i.e., ca. 320 ​K cells/cm^2^) and left to adhere in the incubator for ∼6h. At this point, the Pluronic treatment described below was applied to the bottom channel in the final chip protocol. Next, chip channels were washed gently with pre-warmed B/D complete. The chips were connected to the pre-warmed media-filled fluidic manifolds, “Pod®” (Emulate Inc.). After obtaining fluid connection between chips and Pods, these units were placed in the micro perfusion instrument, “Zoë®” (Emulate Inc.). After finishing the initial regulate cycle program (which pressurizes the medium to increase gas solubility and remove nucleating air bubbles while the system calibrates), the chips were continuously perfused with a flow rate of 30 ​μl/h in both top and bottom channel. Approximately 24h after start of the first regulate cycle, a so-called via wash was performed, dislodging any bubbles in the Pod's reservoirs fluid vials, followed by a second regulate cycle. This was important to minimize the blockage of flow by air bubbles. When following this protocol, no degassing of medium was needed. Approximately 3–5 days after seeding (donor dependent), air-liquid interface (ALI) was established as described in the next paragraph.

#### 5.2.4 Epithelial differentiation at air-liquid interface (ALI) in the airway chip

ALI was established by removing the medium from the top channel and having only medium flowing through the bottom channel. To equalize (hydrostatic) pressure at the membrane level, reservoirs in the Pod connecting the inlet and outlet of the top channel were sealed with 1 ​mL ​B/D complete medium per reservoir. Nonetheless, occasional flooding of the top channel would still occur, requiring daily monitoring. Submerged top channels were emptied manually using a 1000 ​μl pipette. Epithelial cultures were also monitored daily for flow issues and cellular invasion and morphology. If flow in the bottom channel became impaired, chips were disconnected, channels were rinsed with medium, potential air bubbles were dislodged, and chips were re-reconnected after establishing liquid-liquid interface between chip and Pod. If this happened repeatedly in the same sample, the Pod was replaced. Every 48h, medium outlet reservoirs were emptied, and inlet reservoirs were filled with fresh medium.

#### 5.2.5 Application of airflow and stretch

At day 7 of ALI, the flow rate of air in the top channel in AF/S chips was set to ca. 1.2 ​μl/s of airflow. Additionally, the membrane strain rate was set to 5% at a frequency of 0.25 ​Hz.

### 5.3 Invasion scoring

We assigned the following scores to the degree of cell invasion into the bottom channel, as assessed by phase contrast microscopy: 0, no invasion; 1, individual cells in the bottom channel; 2, confluent cell layers in the bottom channel; 3, confluent tissue with multiple layers and/or fibroblast-like cell morphology in the bottom channel.

### 5.4 Prevention of bottom channel invasion

#### 5.4.1 Chemical treatments

Several strategies were tested to assess inhibition of PBEC migration to the bottom channel which are summarized in Supplementary Table 1. Three strategies were included: cell culture media variations, addition of different dose of EC-23 to the currently used medium or addition of dexamethasone at variable concentrations to the cell culture medium. Alternatively, several inhibitors or agonists of signaling pathways were added to the cell culture medium currently used. Neither signaling inhibitors of migration nor altered media composition significantly reduced invasion while preserving differentiation capacity (data not shown).

#### 5.4.2 Hydrogel mechanical barrier

The concurrent seeding of epithelial and endothelial cells onto either side of the membrane can be used as a mechanical barrier to cell migration [35], however, this strategy prevents the study of epithelial cells in isolation and comparison to standard culture conditions. Instead, we tested the use of a mechanical gel barrier. Different compositions of ECM gels in the top channel were tested, including bovine type-1 collagen (FibriCol®, Advanced BioMatrix), Matrigel® (Corning, Corning, NY, USA), Fibronectin (Gibco), collagen IV (Sigma), and the collagen cross-linking agent, microbial transglutaminase (MTG) (Modernist Pantry LLC, Eliot, ME, USA). All ECM mixtures were prepared in PBS- solution and contained the following: collagen I ​= ​0.5 ​mg/mL collagen I; collagen I ​+ ​MTG ​= ​0.5 ​mg/mL collagen I + 4 ​mg/mL MTG; collagen I ​+ ​Fibronectin ​= ​0.5 ​mg/mL collagen I + 200 ​μg/mL Fibronectin; collagen I ​+ ​collagen IV ​= ​0.5 ​mg/mL collagen I + 200 ​μg/mL collagen IV; collagen IV ​+ ​Matrigel® ​= ​400 ​μg/mL collagen IV + 200 ​μg/mL Matrigel®. The gel was prepared by injecting the gel prepolymer solution after membrane surface activation and incubated overnight at 37 ​°C. The next day, the gel was then flushed twice with 100 ​μl of warm medium at 187.5 ​μl/s flow (Eppendorf Xplorer automatic pipet; Eppendorf). This generated a 3D-ECM layer on the membrane with a thickness of 20–85 ​μm, as optimized previously [36] (Supplementary Fig. 7A and B). To assess and confirm ECM scaffold formation of different ECM compositions, we stained the gels with a fluorescent dye. Briefly, 1 ​mg/mL of N-hydroxysuccinimide (NHS) ester dye (Atto 488 NHS Ester, Sigma) was mixed with 50 ​mM borate buffer (pH 9) in 1:500 ratio. Directly after ECM formation, the prepared staining solution was injected into the top channel and the chips were incubated for 25 ​min at room temperature in the dark. The top channel was then rinsed three times with PBS prior to fluorescence imaging. Using ImageJ [37], the ECM-covered area was measured as a percentage to the total channel area via automated thresholding. The entire straight length of the channel was evaluated in all conditions (N ​= ​3 chips each). To measure the ECM thickness, z-stack images with 4 ​μm step size were acquired in two defined regions (left and right part) of the top channel. Using ImageJ, the stacks were 3D-projected and rotated to obtain the 90°- side view of the channel, followed by visualizing the stained ECM with different thicknesses via automated thresholding. Average ECM thickness was measured by calculating the threshold area divided by the length of the image. Basal cells seeded onto the different gel types attached well, and significant reduction in basal channel invasion was observed compared to chips without hydrogels (Supplementary Fig. 1E). At day 14 of ALI a well-differentiated epithelium containing club, ciliated, goblet and basal cells had formed (Supplementary Fig. 7C). However, given substantial variability between ECM lots used for hydrogel preparation and other factors influencing spreading, gelling, and final geometry in the microfluidic channel, it was technically challenging to reliably deposit gels without any holes that allowed for transmigration.

#### 5.4.3 Pluronic anti-adhesion surface treatment

Approximately 6 ​h after hPBEC seeding into the top channel, the bottom channels of all chips were coated with the surfactant Pluronic F-127 (Sigma) to prevent cell adhesion. For this, a minimum of 0.02% w/v Pluronic F-127 was freshly prepared in complete B/D medium and given time to dissolve at room temperature rather than at 37 ​°C, as solubility of Pluronic decreases with temperature [38]. Next, the solution was sterile filtered using a 0.22 ​μm filter. The bottom channel of the chips was emptied and filled with the Pluronic solution, followed by 1h incubation at 37 ​°C. After the incubation, warm medium was used to gently rinse both channels before proceeding with connecting the chips to the perfusion instrument Zoë as described above. Our preliminary data suggest that the bottom channel functionalization can be safely renewed at later timepoints by adding up to 0.2% Pluronic to the perfusion medium for 24–48h, which prevents bottom channel invasion even in highly migratory donors.

### 5.5 Endothelial-epithelial co-culture on chip

Primary Human Lung Microvascular Endothelial Cells (HLMVEC) were purchased from Cell Applications (San Diego, CA, USA) and expanded in Microvascular Endothelial Cell Medium (Cell Applications). Chips were seeded with endothelial cells on day 11 or 12 of ALI. To prepare for endothelial cell seeding, the chips with epithelial tissue in the top channel were disconnected from the Pods. Since Pluronic is thermoreversible and thereby more soluble in medium at lower temperatures [38], we washed the basal chip channels at day 12 of ALI with ice-cold PBS to remove the Pluronic coating. For this, the top channel was filled with warm B/D complete medium, and the bottom channel was incubated three times for 30 ​s, then rinsed, with ice-cold PBS. The bottom channel was filled with 100 ​μg/mL human fibronectin (Sigma) in B/D complete medium. The chips were flipped upside down, leveled and incubated for 1 ​h at 37 ​°C. Just before endothelial cell seeding, cells were washed with PBS and dissociated using Accutase (Sigma), then resuspended at 7.5 ​× ​10^6^ ​cells/mL in endothelial cell medium. The fibronectin solution was removed from the bottom channel and ca. 8 ​μl of the cell suspension was added, resulting in a seeding density of ca. 200, 000 ​cells/cm^2^. The chip was again flipped upside down and left undisturbed in the incubator for 2h. After 2h, the endothelial cell medium was carefully exchanged in the bottom channel. The chip was reconnected to the Pod and brought to ALI using standard procedures. The bottom channel was perfused with a mix of 50% endothelial cell medium and 50% B/D complete medium at standard flow rates (30 ​μl/h).

### 5.6 Airway cell culture inserts

#### 5.6.1 Leiden

To match the seeding conditions on the chips, hPBEC were seeded at high density (150,000 ​cells/well) on ECM-coated 12 ​mm clear polyester cell culture inserts with 0.4 ​μm pore size (Corning Costar, Cambridge, USA, cat.#: 3460). Inserts were coated with 300 ​μg/mL human collagen IV (Sigma) solution in PBS. We also tested a coating mixture consisting of 30 ​μg/mL Purecol (Advanced BioMatrix), 10 ​μg/mL fibronectin (Promocell) and 10 ​μg/mL BSA (ThermoFisher Scientific) in PBS [39,40] but no significant differences in any our endpoints compared to the collagen IV-coated inserts (Supplementary Fig. 8), hence we discontinued this alternative protocol.

Seeded cells were cultured submerged in B/D complete with EC-23 (50 ​nM). When cells were confluent, medium on the top side was removed to initiate differentiation at air-liquid interface. Medium was changed 3 times a week and the top side was washed with warm PBS.

#### 5.6.2 USC

Clear polyester 6.5 ​mm cell culture inserts with 0.4 ​μm pore size (Corning Costar, Cambridge, USA) were pre-coated with 300 ​μg/mL human collagen IV (Sigma) solution in PBS. hPBEC were seeded at high density (70.000 ​cells/well) and cultured as above.

### 5.7 RNA isolation and bulk RNA sequencing

At day 14 of ALI, cells in the airway chip were lysed by disconnecting the chip from the Pod, lodging an empty filter tip at the outlet of the chip, and pipetting 100 ​μl lysis buffer (Promega) with another filter tip into the inlet of the chip. This tip was then lodged into the inlet, such that the two tips inserted at either channel end were capturing the overflowing liquid. When visual inspection indicated lysis of the cells, the lysis solution was collected in a tube. Another 100 ​μl was used to rinse the channel once more, to obtain in total 200 ​μl of lysis solution from one chip. The lysis solution was stored at −20 ​°C until RNA extraction. Total RNA was robotically extracted using the Maxwell tissue RNA extraction kit (Promega) and quantified using the Nanodrop ND-1000 UV–Vis Spectrophotometer (Nanodrop technologies, Wilmington, DE, USA). Next, RNA was sent to GenomeScan (Leiden, the Netherlands) for Bulk RNA sequencing, or converted to cDNA for qPCR analysis. RNA sequencing was performed with the cDNA fragment libraries by the Illumina NovaSeq6000 sequencer using 150 bp pair-end sequencing settings.

### 5.8 RNA sequencing analysis

#### 5.8.1 Data processing

Data processing was performed by GenomeScan. For this, the NEBNext Ultra II Directional RNA Library Prep Kit for Illumina was used to process the samples. The sample preparation was performed according to the protocol “NEBNext Ultra II Directional RNA Library Prep Kit for Illumina” (NEB #E7760 ​S/L). Briefly, mRNA was isolated from total RNA using the oligo-dT magnetic beads. After fragmentation of the mRNA, a cDNA synthesis was performed. This was used for ligation with the sequencing adapters and PCR amplification of the resulting product. The quality and yield after sample preparation were measured with the Fragment Analyzer. The size of the resulting products was consistent with the expected size distribution (a broad peak between 300 and 500 bp). Clustering and DNA sequencing using the NovaSeq6000 was performed according to manufacturer's protocols. A concentration of 1.1 ​nM of DNA was used. Sequence reads were trimmed to remove possible adapter sequences using cutadapt v1.10. Presumed adapter sequences were removed from the read when the bases matched a sequence in the adapter sequence set (TruSeq adapters). For each sample, the trimmed reads were mapped to the human GRCh37.75 reference sequence (Homo_sapiens.GRCh37.75. dna.primary_assembly.fa). The reads were mapped to the reference sequence using a short-read aligner based on Burrows-Wheeler Transform (Tophat v2.0.14) with default settings. Based on the mapped locations in the alignment file the frequency of how often a read was mapped on a transcript was determined with HTSeq v0.11.0. The hit counts were summarized and reported using the gene_id feature in the annotation file. Only unique reads that fall within exon regions were counted. To enable comparison of gene/transcript expression across all samples outside of the context of differential expression analysis, RPKM/FPKM (reads/fragments per kilobase of exon per million reads mapped), TPM (transcripts per million), and CPM values were calculated with Cufflinks v2.2.1.

#### 5.8.2 Differential expressed genes

The read counts were used in the DESeq2 v2-1.14 package, a statistical package within the R platform R v3.3.0. The differentially expressed genes were identified based on read counts of genes >10, with a log2 fold change of >1.5 and q value of <0.05. Since groups had a small sample size and over-dispersion, an expression curve model based on a negative binomial distribution and local regression was used to estimate the relationship between the mean and variance of each gene. For heat maps, log2 expression of read counts are normalized with Z scores calculated by: (X (value of the individual sample) - μ (average of the row))/σ (standard deviation of the row).

#### 5.8.3 Gene set analysis

The gene sets of differentially expressed genes were analyzed in GSEA (Geneset Enrichment analysis) which also shows the related pathways. The significantly up- and downregulated genes (False Discovery Rate (FDR) ​< ​0.05) and related pathways between CTL and AF/S chips and between CTL chip and COLIV insert are provided in Table 2 and Supplementary Tables 2 and 3

### 5.9 cDNA and qPCR

For cDNA conversion, 500 ​ng of total RNA was reverse transcribed using oligo dT primers (Qiagen Benelux B·V., Venlo, The Netherlands) and M-MLV Polymerase (Thermo Fisher Scientific) at 42 ​°C. All quantitative PCR (qPCR) reactions were performed in triplicate on a CFX-384 Real-Time PCR detection system (Bio-Rad Laboratories, Veenendaal, The Netherlands), using primers shown in Supplementary Table 5 and IQ SYBRGreen supermix (Bio-Rad). The relative standard curve method was used to calculate arbitrary gene expression using CFX-manager software (Bio-Rad). Two reference genes were included to calculate the normalized gene expression.

### 5.10 CXCL8 ELISA

The protein encoded by CXCL8 (interleukin-8 or IL-8), was measured with use of the Recombinant Human IL-8/CXCL8 Protein ELISA kit of R&D (Abingdon, UK) according to manufacturer's instructions and analyzed with use of 4-parameter logistic curve fitting in Prism 8 (version 8.1.1).

### 5.11 Multiplex protein measurements

Cytokine and growth factor secretion was measured with the Bio-Plex Pro Human Cytokine Screening Panel (Bio-Rad #M500KCAF0Y), a 27-plex assay. Effluent culture medium was collected from the chips (collected in the Pod's effluent reservoir) from cultures with 8 different donors. These samples were diluted 1:4 in assay buffer provided with the kit. Manufacturer's instructions were followed to perform this assay, with minor adjustments to the protocol; one third of the proposed number of magnetic beads and detection antibodies was used. Plate readout was performed on a Luminex 100/200 System (Luminex, ‘s-Hertogenbosch, The Netherlands).

### 5.12 IF staining and histology

At 14 day of ALI, cells in the Chip were fixed using 4% paraformaldehyde (PFA) solution. The PFA solution was pipetted into both channels and incubated for 20 ​min at RT, washed with PBS and stored in PBS at 4 ​°C until staining. Chips were subsequently removed from the carrier and cells were blocked and permeabilized using 0.5% (v/v) Triton-X 100 in PBS with 5% BSA for 60 ​min at RT. Chips were cut into 2 pieces using a razor blade and channels were filled with primary antibodies (Supplementary Table 6) diluted in the Triton/BSA buffer and incubated for 1h at RT. Samples were rinsed three times 5 ​min with PBS. Next, channels were filled with secondary antibodies diluted in Triton/BSA buffer which was left incubating for 1 ​h at RT, followed by a triple 5 ​min wash with PBS. 4′,6-Diamidino-2-phenylindole (DAPI, Sigma) was used to stain cell nuclei. Channels were next filled with prolong gold anti-fade (Thermo Scientific) and stored in the dark at 4 ​°C until imaging. Chips were imaged on Bellco coverslips, 25 ​× ​75mm (Electron Microscopy Sciences, Hatfield, PA, SUA) with 0.13–0.17 ​mm thickness using a Leica DMi8 microscope equipped with an Andor Dragonfly 200 spinning disk confocal using a 10× objective (NA 0.30×), 20× water objective (NA 0.50) or 40× water objective (NA 0.80).

### 5.13 Cross-sectional imaging of thin chip slices

A new sectioning technique was developed to obtain cross-sectional slices of the chip for histology and immunofluorescence staining. Chips were fixed as described as above. The PDMS surrounding the chip channel was cut away as much as possible using a razor blade. Next, the chip channel was mounted on the specimen tube using glue (Pattex instant glue) of a semi-automated vibrating microtome (Compresstome® VF-300-0Z, CMA, Kista, Sweden) and placed in the tube holder. Next, 150 ​μm thick slices were cut and collected in PBS-filled wells of a 12-well plate. Slices were stained in the well for classical H&E or immunofluorescence as described above. Slices were imaged on bellco coverslips as described above for the complete chip.

### 5.14 IF image processing and quantifications

#### 5.14.1 Cell type quantification

Raw images were split into individual channels, median filtered, cropped and CLAHE-filtered [41] to remove illumination artifacts, and auto-thresholded to retrieve the area fraction positive for each cell type marker. Semi-automated batch processing was conducted using ImageJ-Fiji [42].

#### 5.14.2 VANGL1 crescent quantification

Raw images were split into individual channels and cropped as needed to remove illumination artifacts. We used a Matlab script to de-noise the images with Gaussian, median, and Wiener filters and detected the VANGL1 crescents with Hessian based multiscale filtering (Supplementary Fig. 9). The binarized crescents were skeletonized to yield lines of single pixel width and compute their total length. VANGL1 crescent density (D_C_) was defined as $D_{C}=\sum L_{i}\;/A$, where A is the area of the field of view in μm^2^, L_i_ is the length of VANGL1 crescent *i* in μm, and the sum ranges over all *i* ​= ​1 … N crescents detected in the field of view. For each donor, final crescent density was reported as the average value from all fields of view.

### 5.15 Measurements of CBF and MCC

#### 5.15.1 Ciliary beat frequency measurement

CBF was measured using high-speed video microscopy followed by signal processing of the recordings as described previously in detail [12]. Briefly, to optimize optical imaging quality, inserts were washed for 10 ​min with PBS and the returned to ALI whereas Lung-Chips kept submerged in PBS or culture medium during imaging. Samples were imaged using a Leica DMi8 microscope equipped with phase-contrast objectives and a PCO Edge 4.2 high-speed camera with camera link interface that was operated from a PC desktop computer using the ImageJ-Fiji [42] with Micro-Manager [43]. Per sample, 6 to 8 fields of view (166 ​× ​166 μm, with ca. 3 pixel/μm spatial resolution) were recorded under Koehler illumination at a temporal resolution of 200 frames per second. Using a custom algorithm in Matlab (MathWorks), regions containing ciliary beat activity were detected automatically by computing the standard deviation of each pixel's intensity over time, which reveals motion, and thresholding the resulting image using Otsu's method to segment area with detected motion. Ciliary beat density was computed by dividing the number of pixels with detected motion by the total number of pixels in the image. In regions with ciliary beat, the dominant beat frequency was identified from the smoothed pixel intensity over time using Fast Fourier Transform (FFT). The ciliary beat frequencies and ciliary beat densities measured in all movies per sample were averaged for further statistical analysis.

#### 5.15.2 Mucociliary clearance measurement

MCC was measured by adding fluorescent tracer particles to the top of samples, recording the movement of the tracers over time using video microscopy, and tracking the displacement of the particles in the resulting videos using specialized software to derive transport velocities. Briefly, the samples were washed and prepared as above before adding 30 ​μl of a 1:200 dilution of 1-μm sized fluorescent polystyrene microspheres (ThermoFisher Scientific) in PBS. Samples were imaged using a Leica DMi8 microscope equipped with a fluorescence light source and appropriate filters. Per sample, 3–6 fields of view (ca. 1200 ​× ​1200 μm each) were recorded for 10–20 ​s at a temporal resolution of 30 frames per second. Using a batch-processing script in ImageJ-Fiji with the Trackmate plugin [44], we tracked the bead displacement per time step to derive their trajectories and flow speed. To identify and filter out potential background flow, we used a supervised machine learning approach in Matlab to classify trajectories as either background or true signal. Briefly, we imaged bead movements in cell-free chips and inserts as well as in strong MCC flow. To generate the ground truth data, we labeled ca. 40,000 trajectories correctly as either true signal (MCC) or background flow. To characterize the trajectories, we measured the following metrics: mean speed, median speed, standard deviation of speed, path length, Euclidian distance covered, the ratio of Euclidian distance covered to path length (directness), mean flow angle, number of nodes, and maximal acceleration. Using the Matlab classification learner app, we trained a decision tree model with 5-fold cross-validation to identify true and background trajectories. We found that Euclidian distance, median speed, and directness below a threshold level were sufficient indicators to classify trajectories as background flow with an accuracy of more than 95%. This simple classification strategy was then used to automatically filter out the background flow in all data sets (Supplementary Fig. 10A). Matlab was used to further analyze the flow as described below.

#### 5.15.3 MCC polar order parameter

The main direction of each bead trajectory was determined by the measuring the angle ${Ɵ}_{i}$ formed between the channel axis and the line between start point and end point of each bead trajectory (Fig. 2D), where *i* ranges from 1 to the number of trajectories per sample. For each sample, an average unit vector $\hat{u}$ was determined from all measured angles: $\hat{u}=\left\langle \left[\sin\left({Ɵ}_{i}\right),\cos\left({Ɵ}_{i}\right)\right]\right\rangle$, where the outer brackets indicate averaging. The PO was computed by dotting the average unit vector with the unit vector $\hat{n}$ pointing towards the channel inlet: $PO=\hat{u}∙\hat{n}$. The PO hence ranges from −1 to 1, with −1 and 1 indicating perfect alignment of the mean flow vector towards the channel outlet or inlet, respectively. Intermediate values indicate partial alignment towards one the channel ends, i.e., negative values towards outlet and positive values towards inlet. A PO near 0 indicates isotropic flow or alignment of the mean flow vector towards the walls. Trajectory data from all movies per sample were pooled for the analysis. Therefore, alternating, perpendicular, and random flow without any bias would all be expected to result in an average PO of zero.

#### 5.15.4 MCC coverage and speed

To measure MCC coverage and area-averaged speed, the trajectories were converted into a Eulerian velocity vector field $U\left(x,y\right)=u\left(x,y\right)\hat{i}+v\left(x,y\right)\hat{j}$ by averaging the flow velocity component in x direction (u) and in y direction (v) measured at each coordinate point over time (since multiple beads may pass over the same location) (Supplementary Fig. 10B left and center). The Matlab “alphaShape” function was used to generate coherent areas from these point measurements and estimate MCC coverage $C_{MCC}$, i.e., the fraction of surface area with measurable MCC: $C_{MCC}=\frac{A_{MCC}}{A_{Total}}$ , where $A_{MCC}$ is the area with measurable MCC and $A_{Total}$ is the total tissue surface area visible in the field of view (Supplementary Fig. 10C right). Area-averaged speed $\bar{M}$ was computed by weighting the average flow speed in active regions by their relative surface fraction: $\bar{M}=C_{MCC}\left\langle \left|U\right|\right\rangle$, where |U| is the scalar field of flow magnitudes and the brackets indicate averaging. Flow data from all movies per sample were pooled for statistical analysis.

### 5.16 Statistical methods

Statistical analysis was conducted using GraphPad Prism version 8 and 9 (GraphPad Software Inc). Differences were considered significant at p ​< ​0.05. Statistical significance, statistical methods, and the number of donors and samples used are detailed in the figures and figure legends. Where number of donors is reported, this always refers to the number of different donors.

## Funding

The work at 10.13039/501100005039LUMC is supported by an 10.13039/100006939EU 10.13039/501100000654Marie Curie Global Fellowship (#748569), the Dutch Society for the Replacement of Animal Testing (Stichting Proefdiervrij), the Netherlands Organization for Health Research and Development (ZonMw; #114021508) and the 10.13039/501100015084Lung Foundation Netherlands (grant #4.1.19.021). ALR is supported by the 10.13039/100015603Hastings Foundation, the Tyler Health and Education Trust and 10.13039/100000897Cystic Fibrosis Foundation (grants #FIRTH17XX0 and #FIRTH21XX0). JCN is supported by an ERC-STG (MecCOPD # 959219). The funding sources had no involvement in collection, analysis and interpretation of data; in the writing of the report; and in the decision to submit the article for publication.

## Credit author statement

Janna C. Nawroth: Conceptualization, Methodology, Formal analysis, Investigation, Data curation, Writing – original draft, Writing – review & editing, Visualization, Supervision. Doris Roth: Conceptualization, Methodology, Investigation, Writing – review & editing, Visualization, Funding acquisition. Annemarie van Schadewijk: Methodology, Investigation, Visualization. Abilash Ravi: Formal analysis, Writing – review & editing, Visualization. Tengku Ibrahim Maulana: Methodology, Investigation, Writing – review & editing, Visualization. Christiana N. Senger: Methodology, Investigation. Sander van Riet: Formal analysis, Investigation, Writing – review & editing, Visualization. Dennis K. Ninaber: Investigation. Amy M. de Waal: Investigation. Dorothea Kraft: Investigation. Pieter S. Hiemstra: Resources, Writing – review & editing, Supervision, Funding acquisition. Amy L Ryan: Resources, Writing – review & editing, Supervision, Funding acquisition. Anne M. van der Does: Conceptualization, Methodology, Formal analysis, Investigation, Writing – original draft, Writing – review & editing, Visualization, Supervision, Project administration, Funding acquisition.

## Declaration of competing interest

The authors declare the following financial interests/personal relationships which may be considered as potential competing interests:

Anne M. van der Does reports financial support was provided by 10.13039/501100000780European Union. Janna C. Nawroth reports financial support and equipment, drugs, or supplies were provided by Emulate Inc. Doris Roth reports equipment, drugs, or supplies was provided by Emulate Inc. Anne M. van der Does reports equipment, drugs, or supplies was provided by Emulate Inc. Tengku Ibrahim Maulana reports equipment, drugs, or supplies was provided by Emulate Inc. Janna C. Nawroth reports financial support was provided by 10.13039/501100000780European Union. Janna C. Nawroth has patent #US20220106547A1 pending to Emulate Inc. J.N. is a former employee of Emulate, Inc. A.D. was supported by a Global 10.13039/501100000654Marie Curie fellowship (No. 748569) that included a 1-yr visit (2018–2019) at Emulate Inc. to work on their Lung-Chips. D.R. and T.M. performed their internship at Emulate Inc in 2018 and 2019, respectively. Materials from Emulate Inc. Related to this work were therefore provided by Emulate Inc. The remaining authors have no conflicts of interest relevant to this publication.

## Data Availability

Data will be made available on request.

## References

[1] Bustamante-Marin X.M., Ostrowski L.E. (2017). Cilia and mucociliary clearance. Cold Spring Harbor Perspect. Biol..

[2] Oltean A., Schaffer A.J., Bayly P.V., Brody S.L. (2018). Quantifying ciliary dynamics during assembly reveals stepwise waveform maturation in airway cells. Am. J. Respir. Cell Mol. Biol..

[3] Saint-Criq V., Delpiano L., Casement J., Onuora J.C., Lin J., Gray M.A. (2020). Choice of differentiation media significantly impacts cell lineage and response to CFTR modulators in fully differentiated primary cultures of cystic Fibrosis human airway epithelial cells. Cells.

[4] Leung C., Wadsworth S.J., Yang S.J., Dorscheid D.R. (2020). Structural and functional variations in human bronchial epithelial cells cultured in air-liquid interface using different growth media. Am. J. Physiol. Lung Cell Mol. Physiol..

[5] Yang J., Zuo W.-L., Fukui T., Chao I., Gomi K., Lee B., Staudt M.R., Kaner R.J., Strulovici-Barel Y., Salit J., Crystal R.G., Shaykhiev R. (2017). Smoking-dependent distal-to-proximal repatterning of the adult human small airway epithelium. Am. J. Respir. Crit. Care Med..

[6] Wirtz H.R., Dobbs L.G. (2000). The effects of mechanical forces on lung functions. Respir. Physiol..

[7] LaPrad A.S., Lutchen K.R., Suki B. (2013). A mechanical design principle for tissue structure and function in the airway tree. PLoS Comput. Biol..

[8] Tsega E.G. (2018). Computational fluid dynamics modeling of respiratory airflow in tracheobronchial airways of infant, child, and adult. Comput. Math. Methods Med..

[9] Sidhaye V.K., Schweitzer K.S., Caterina M.J., Shimoda L., King L.S. (2008). Shear stress regulates aquaporin-5 and airway epithelial barrier function. Proc. Natl. Acad. Sci. USA.

[10] Sanchez-Esteban J., Cicchiello L.A., Wang Y., Tsai S.W., Williams L.K., Torday J.S., Rubin L.P. (2001). Mechanical stretch promotes alveolar epithelial type II cell differentiation. J. Appl. Physiol..

[11] Huh D., Matthews B.D., Mammoto A., Montoya-Zavala M., Hsin H.Y., Ingber D.E. (2010). Reconstituting organ-level lung functions on a chip. Science.

[12] Benam K.H., Novak R., Nawroth J., Hirano-Kobayashi M., Ferrante T.C., Choe Y., Prantil-Baun R., Weaver J.C., Bahinski A., Parker K.K., Ingber D.E. (2016). Matched-comparative modeling of normal and diseased human airway responses using a microengineered breathing lung chip. Cell Systems.

[13] Nawroth J.C., Lucchesi C., Cheng D., Shukla A., Ngyuen J., Shroff T., Varone A., Karalis K., Lee H.-H., Alves S., Hamilton G.A., Salmon M., Villenave R. (2020). A microengineered airway lung chip models key features of viral-induced exacerbation of asthma. Am. J. Respir. Cell Mol. Biol..

[14] Si L., Bai H., Rodas M., Cao W., Oh C.Y., Jiang A., Moller R., Hoagland D., Oishi K., Horiuchi S., Uhl S., Blanco-Melo D., Albrecht R.A., Liu W.-C., Jordan T., Nilsson-Payant B.E., Golynker I., Frere J., Logue J., Haupt R., McGrath M., Weston S., Zhang T., Plebani R., Soong M., Nurani A., Kim S.M., Zhu D.Y., Benam K.H., Goyal G., Gilpin S.E., Prantil-Baun R., Gygi S.P., Powers R.K., Carlson K.E., Frieman M., tenOever B.R., Ingber D.E. (2021). A human-airway-on-a-chip for the rapid identification of candidate antiviral therapeutics and prophylactics. Nat Biomed Eng.

[15] Tan J.L., Liu W., Nelson C.M., Raghavan S., Chen C.S. (2004). Simple approach to micropattern cells on common culture substrates by tuning substrate wettability. Tissue Eng..

[16] Crystal R.G., Randell S.H., Engelhardt J.F., Voynow J., Sunday M.E. (2008). Airway epithelial cells. Proc. Am. Thorac. Soc..

[17] Amatngalim G.D., Schrumpf J.A., Dishchekenian F., Mertens T.C.J., Ninaber D.K., van der Linden A.C., Pilette C., Taube C., Hiemstra P.S., van der Does A.M. (2018). Aberrant epithelial differentiation by cigarette smoke dysregulates respiratory host defence. Eur. Respir. J..

[18] Sul B., Wallqvist A., Morris M.J., Reifman J., Rakesh V. (2014). A computational study of the respiratory airflow characteristics in normal and obstructed human airways. Comput. Biol. Med..

[19] Horani A., Ferkol T.W. (2018). Advances in the genetics of primary ciliary dyskinesia: clinical implications. Chest.

[20] Vladar E.K., Nayak J.V., Milla C.E., Axelrod J.D. (2016). Airway epithelial homeostasis and planar cell polarity signaling depend on multiciliated cell differentiation. JCI Insight.

[21] Duclos G.E., Teixeira V.H., Autissier P., Gesthalter Y.B., Reinders-Luinge M.A., Terrano R., Dumas Y.M., Liu G., Mazzilli S.A., Brandsma C.-A., van den Berge M., Janes S.M., Timens W., Lenburg M.E., Spira A., Campbell J.D., Beane J. (2019). Characterizing smoking-induced transcriptional heterogeneity in the human bronchial epithelium at single-cell resolution. Sci. Adv..

[22] Ruiz García S., Deprez M., Lebrigand K., Cavard A., Paquet A., Arguel M.-J., Magnone V., Truchi M., Caballero I., Leroy S., Marquette C.-H., Marcet B., Barbry P., Zaragosi L.-E. (2019). Novel dynamics of human mucociliary differentiation revealed by single-cell RNA sequencing of nasal epithelial cultures. Development.

[23] Rock J.R., Randell S.H., Hogan B.L.M. (2010). Airway basal stem cells: a perspective on their roles in epithelial homeostasis and remodeling. Disease Models & Mechanisms.

[24] Watson J.K., Rulands S., Wilkinson A.C., Wuidart A., Ousset M., Van Keymeulen A., Göttgens B., Blanpain C., Simons B.D., Rawlins E.L. (2015). Clonal dynamics reveal two distinct populations of basal cells in slow-turnover airway epithelium. Cell Rep..

[25] Gsell S., Loiseau E., D'Ortona U., Viallat A., Favier J. (2020). Hydrodynamic model of directional ciliary-beat organization in human airways. Sci. Rep..

[26] Ramirez-San Juan G.R., Mathijssen A.J.T.M., He M., Jan L., Marshall W., Prakash M. (2020). Multi-scale spatial heterogeneity enhances particle clearance in airway ciliary arrays. Nat. Phys..

[27] Elgeti J., Gompper G. (2013). Emergence of metachronal waves in cilia arrays. Proc. Natl. Acad. Sci. U.S.A..

[28] Hill D.B., Swaminathan V., Estes A., Cribb J., O'Brien E.T., Davis C.W., Superfine R. (2010). Force generation and dynamics of individual cilia under external loading. Biophys. J..

[29] Wallingford J.B. (2010). Planar cell polarity signaling, cilia and polarized ciliary beating. Curr. Opin. Cell Biol..

[30] Sone N., Konishi S., Igura K., Tamai K., Ikeo S., Korogi Y., Kanagaki S., Namba T., Yamamoto Y., Xu Y., Takeuchi K., Adachi Y., Chen-Yoshikawa T.F., Date H., Hagiwara M., Tsukita S., Hirai T., Torisawa Y.-S., Gotoh S. (2021). Multicellular modeling of ciliopathy by combining iPS cells and microfluidic airway-on-a-chip technology. Sci. Transl. Med..

[31] Yi B., Xu Q., Liu W. (2022). An overview of substrate stiffness guided cellular response and its applications in tissue regeneration. Bioact. Mater..

[32] Greaney A.M., Adams T.S., Brickman Raredon M.S., Gubbins E., Schupp J.C., Engler A.J., Ghaedi M., Yuan Y., Kaminski N., Niklason L.E. (2020). Platform effects on regeneration by pulmonary basal cells as evaluated by single-cell RNA sequencing. Cell Rep..

[33] Nawroth J.C., Barrile R., Conegliano D., van Riet S., Hiemstra P.S., Villenave R. (2018). Stem cell-based Lung-on-Chips: the best of both worlds?. Adv. Drug Deliv. Rev..

[34] Peters-Hall J.R., Coquelin M.L., Torres M.J., LaRanger R., Alabi B.R., Sho S., Calva-Moreno J.F., Thomas P.J., Shay J.W. (2018). Long-term culture and cloning of primary human bronchial basal cells that maintain multipotent differentiation capacity and CFTR channel function. Am. J. Physiol. Lung Cell Mol. Physiol..

[35] Si L., Bai H., Rodas M., Cao W., Oh C.Y., Jiang A., Moller R., Hoagland D., Oishi K., Horiuchi S., Uhl S., Blanco-Melo D., Albrecht R.A., Liu W.-C., Jordan T., Nilsson-Payant B.E., Golynker I., Frere J., Logue J., Haupt R., McGrath M., Weston S., Zhang T., Plebani R., Soong M., Nurani A., Kim S.M., Zhu D.Y., Benam K.H., Goyal G., Gilpin S.E., Prantil-Baun R., Gygi S.P., Powers R.K., Carlson K.E., Frieman M., tenOever B.R., Ingber D.E. (2021). A human-airway-on-a-chip for the rapid identification of candidate antiviral therapeutics and prophylactics. Nature Biomedical Engineering.

[36] Nawroth J.C., Petropolis D.B., Manatakis D.V., Maulana T.I., Burchett G., Schlünder K., Witt A., Shukla A., Kodella K., Ronxhi J., Kulkarni G., Hamilton G., Seki E., Lu S., Karalis K.C. (2021). Modeling alcohol-associated liver disease in a human Liver-Chip. Cell Rep..

[37] Abramoff M., Magelhaes P., Ram S. (2004). Image processing with ImageJ. Biophot. Int..

[38] Escobar-Chávez J.J., López-Cervantes M., Naïk A., Kalia Y.N., Quintanar-Guerrero D., Ganem-Quintanar A. (2006). Applications of thermo-reversible pluronic F-127 gels in pharmaceutical formulations. J. Pharm. Pharmaceut. Sci..

[39] Wang Y., Ninaber D.K., van Schadewijk A., Hiemstra P.S. (2020). Tiotropium and fluticasone inhibit rhinovirus-induced mucin production via multiple mechanisms in differentiated airway epithelial cells. Front. Cell. Infect. Microbiol..

[40] van Riet S., van Schadewijk A., de Vos S., Vandeghinste N., Rottier R.J., Stolk J., Hiemstra P.S., Khedoe P. (2020). Modulation of airway epithelial innate immunity and wound repair by M(GM-CSF) and M(M-CSF) macrophages. J. Innate Immun..

[41] Zuiderveld K. (1994). Contrast limited adaptive histogram equalization. Graphics Gems.

[42] Schindelin J., Arganda-Carreras I., Frise E., Kaynig V., Longair M., Pietzsch T., Preibisch S., Rueden C., Saalfeld S., Schmid B., Tinevez J.-Y., White D.J., Hartenstein V., Eliceiri K., Tomancak P., Cardona A. (2012). Fiji: an open-source platform for biological-image analysis. Nat. Methods.

[43] Edelstein A., Amodaj N., Hoover K., Vale R., Stuurman N. (2010). Computer control of microscopes using μManager. Curr. Protoc. Mol. Biol..

[44] Jaqaman K., Loerke D., Mettlen M., Kuwata H., Grinstein S., Schmid S.L., Danuser G. (2008). Robust single-particle tracking in live-cell time-lapse sequences. Nat. Methods.

